# Ball-Milled Spent Coffee Ground Biochar Effectively Removes Caffeine from Water

**DOI:** 10.3390/w17060881

**Published:** 2025-03-19

**Authors:** Yicheng Yang, Yongshan Wan, Jianjun Chen, Hao Chen, Yuncong Li, Rafael Muñoz-Carpena, Yulin Zheng, Jinsheng Huang, Yue Zhang, Bin Gao

**Affiliations:** 1 Department of Agricultural and Biological Engineering, University of Florida, Gainesville, FL 32611, USA;; 2 US EPA Center for Environmental Measurement and Modeling, Gulf Breeze, FL 32561, USA; 3 Mid-Florida Research & Education Center, University of Florida, Apopka, FL 32703, USA;; 4 Department of Agriculture, Landscape, and Environment, University of Vermont, Burlington, VT 05405, USA;; 5 Department of Soil and Water Sciences, Tropical Research and Education Center, Institute of Food and Agricultural Sciences, University of Florida, Homestead, FL 33031, USA;; 6 Department of Civil and Environmental Engineering, Rensselaer Polytechnic Institute, Troy, NY 12180, USA;

**Keywords:** ball milling, biochar, adsorption, spent coffee ground, caffeine, completely mixed flow reactor

## Abstract

Caffeine in aquatic ecosystems is an emerging contaminant causing significant environmental concern. In this work, spent coffee ground (SCG) was pyrolyzed at 300, 450, and 600 °C to produce pristine SCG biochars (CG), which were then ball-milled to produce ball-milled SCG biochars (BMCG). A batch experiment with ball-milled and pristine biochars showed that ball-milled biochars pyrolyzed at 450 °C and 600 °C had the highest capacities to adsorb caffeine. Subsequently, ball-milled CG450 (BMCG450) was selected for further analysis. The results showed that ball milling dramatically augmented the specific surface area and oxygen-containing functional groups of the biochar. The Langmuir maximum caffeine adsorption capacity was 82.65 mg/g. Both solution pH and ionic strength affected caffeine removal by BMCG450. As pH increased, increased electrostatic repulsion limited caffeine adsorption onto the biochar. However, an increase in ion strength slightly enhanced caffeine adsorption because of the electrostatic screening effect of cations. The ball-milled SCG biochar also showed high adsorption efficiency in a completely mixed flow reactor under continuous flow conditions. Our study indicates that ball-milled SCG biochar at 450 °C can serve as a viable sorbent for the removal of caffeine from water.

## 1. Introduction

**Figure 1.**
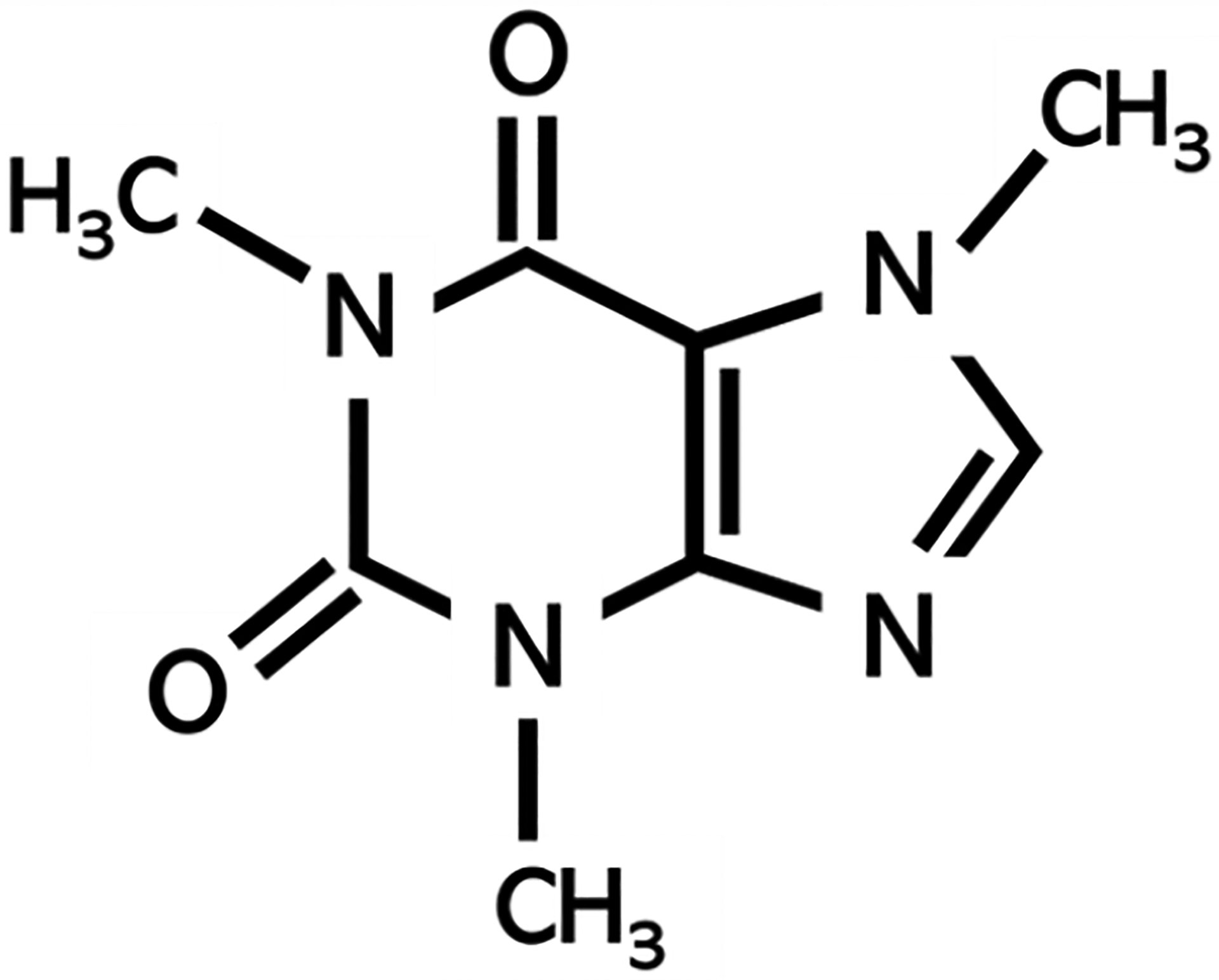
Chemical structure of caffeine.

Caffeine (1, 3, 7-trimethylxanthine) is an emerging contaminant that has attracted widespread attention because it has been detected in many water bodies, with potential risks to ecosystems and public health. Caffeine, an alkaloid of the methylxanthine family (Figure 1), naturally exists in coffee, tea, and cocoa beans [1]. Nowadays, caffeine, as a food additive, is used to produce different foods and beverages, such as energy drinks, chewing gum, and candies [2]. Caffeine can stimulate the human central nervous system and reduce drowsiness; therefore, it is consumed all over the world.

The average daily caffeine consumption varies in different countries, and the worldwide average is about 70 mg per person [3]. In the USA, Drewnowski and Rehm [4] found that adults consume 173 mg of caffeine per person per day on average, while in Europe, the daily caffeine consumption ranges from 137 to 275 mg per person [5]. Meanwhile, spent coffee ground (SCG), as the primary waste produced in the coffee industry, annually reaches approximately 60 million tons globally [6]. Although SCG is a carbon-rich material, it is often labeled as waste and generally disposed of, becoming an environmental burden. The main portion of ingested caffeine is metabolized by the human liver, and about 3% of caffeine enters the sewage system by urine excretion [7]. Moreover, the disposal of caffeine-containing medicine from hospitals and unconsumed caffeine-containing products from restaurants and households also inputs caffeine into the water environment through sewage systems and landfill leaching.

Caffeine is detected in different types of water bodies, including rivers (14.5~127,092 ng/L), lakes (6~250 ng/L), seawater (5.2~1528.2 ng/L), and groundwater (88 ng/L) [8–14]. The effect of caffeine on species living in different types of aquatic ecosystems has been investigated. Gaudet-Hull et al. [15] found that the development of *Xenopus laevis* egg was affected by caffeine in the water. Moore et al. [16] pointed out that caffeine could impair the reproduction of *Ceriodaphnia dubia* and inhibit the growth of *Pimephales promelas*. Besides affecting freshwater species, caffeine could also negatively impact marine organisms, including algae, Bivalvia, and polychaeta. Aguirre-Martínez et al. [17] showed that caffeine caused growth inhibition of *Isochrysis galbana*. Capolupo et al. [18] illustrated that caffeine exposure could increase the oxidative stress of *Ruditapes philippinarum*. Pires et al. [19] found that long-term caffeine exposure could affect the regenerative capacity of *Diopatra neapolitana*.

Various technologies, such as adsorption [20], advanced oxidative processes [21], biodegradation [22], and membrane separation [23] have been developed to remove caffeine from water. A catalytic MnO_2_/MXene/chitosan nanocomposite nanofiltration membrane was fabricated and had a remarkable caffeine removal rate of 99.9% [24]. Under optimized environmental conditions, the fungus *Trametes versicolor* can achieve a caffeine degradation efficiency of up to 98% [25]. In addition, activated carbon derived from artichoke leaves was used as an adsorbent, with a maximum adsorption capacity of 290.86 mg/g [26]. Among the available technologies, adsorption is considered as a practical and feasible approach because it is affordable, highly efficient, and user-friendly, with no sludge formation [27]. Several adsorbents, especially novel carbonaceous material adsorbents with porous structures, have been examined to determine their performance in the sorption of caffeine [28–30].

Biochar is a carbonaceous adsorbent produced by the pyrolysis of biomass under an oxygen-limited environment [31]. Biochar has a high specific surface area and porosity with diverse functional groups, serving as a viable sorbent for the removal of contaminants from water [32]. Several engineering modifications have been applied to biochar, such as surface oxidation [33], acid/base treatment [34], and surface coating [35], to further enhance its adsorption capacity. Most of these modifications are chemical-based, requiring additional chemical reagents, which results in extra costs and the formation of by-products. Ball milling, different from most modifications mentioned, is an environment-friendly and effective physical treatment. It mechanically turns biochar particles into microscale- or nanoscale-size powder with low energy consumption, resulting in increases in its specific surface area (SSA) and oxygen-containing functional groups [36,37]. Previous studies have investigated the effects of ball milling on biochar’s performance in the adsorption of organic pollutants. Huang, Zimmerman, Chen, and Gao [31] indicated that ball milling increased the sulfamethoxazole removal capacity of hickory biochar by up to 83.3%. Zhang et al. [38] applied ball milling to enhance the adsorption of synthetic musk by wheat straw biochar. They found that, after ball milling, biochar’s adsorption capacity for synthetic musk increased dramatically from 609 mg/kg to 2098 mg/kg. However, so far, there has been no research on the application of ball-milled biochar, especially biochar derived from SCG, in removing caffeine from water. Furthermore, current research on the removal of caffeine by biochar has only focused on batch studies to evaluate biochar’s adsorption capacities. To obtain a better understanding of biochar’s performance under more practical circumstances, continuously flowing reactors—such as completely mixed flow reactors (CMFRs)—need to be applied as a method for caffeine removal. CMFRs offer several advantages, including a continuous capacity for removing contaminants, a consistent concentration of contaminants in the effluent, and a constant level of removal efficiency [39].

In this study, we investigated the adsorption of ball-milled SCG biochar for caffeine removal, along with the key influencing factors affecting caffeine adsorption. Additionally, we explored the adsorption mechanisms to better understand the interactions between caffeine and the biochar. Finally, we evaluated caffeine removal efficiency under dynamic conditions in a CMFR. The novelty of this study lies in the utilization of SCG, a byproduct of the coffee production process, to synthesize biochar for the adsorption of caffeine, an emerging contaminant in aquatic environments. This approach effectively addresses two environmental challenges within the coffee industry: (1) the management of SCG and (2) the mitigation of caffeine-induced water pollution. By integrating waste reusage with pollutant removal, this study aims to provide a sustainable and circular solution, achieving a ‘two birds with one stone’ effect.

## 2. Materials and Methods

### 2.1. Materials

SCG was collected from home kitchens and school break rooms. It was dried at 80 °C to remove moisture prior to biochar production. Caffeine was purchased from Thermo Scientific (Waltham, MA, USA). A caffeine stock solution (500 mg/L) was prepared and then diluted for subsequent experiments using deionized (DI) water (Thermo Scientific Barnstead Nanopure). The caffeine solution was processed by sonication and then stored at 25 °C. All required chemicals were of analytical grade.

### 2.2. Biochar Production and Ball Milling

The pristine SCG biochar was produced following a similar process of previous studies [40,41]. Briefly, the dried SCG was placed in an N_2_-filled tubular furnace (Kejia Furnace KJ-T1200, Zhengzhou, China) and pyrolyzed at a heating rate of 10 °C/min to three different final temperatures (300, 450, and 600 °C); each temperature was held for 1 h. The SCG biochar samples were labeled based on pyrolysis temperature (e.g., CG300 for SCG biochar at 300 °C). The yield percentage of the final product at each pyrolysis temperature was as follows: CG300 yielded 75.29%, CG450 32.41%, and CG600 26.38%.

To produce ball-milled SCG biochar samples, 1.8 g of each pristine biochar was mixed with 180 g of grinding agate balls (diameter = 6 mm) in a 500 mL agate jar. The jars were subsequently placed within a planetary ball milling machine (Across International PQ-N2, Livingston, NJ, USA) operated at 300 rpm for 12 h, with a change in rotation direction every 3 h. The ball-milled biochar was identified by adding the prefix ‘BM’ to the labels (e.g., BMCG300 for SCG biochar at 300 °C with ball-milling treatment).

### 2.3. Batch Adsorption Experiment

The biochar screening experiment was conducted at room temperature (25 ± 0.5 °C) by adding 100 mg of pristine or ball-milled biochar into 50 mL of a 100 mg/L caffeine solution in a conical centrifuge tube (Thermo Scientific Nunc, Waltham, MA, USA). The tubes were placed on a mechanical shaker at 200 rpm for 24 h. Samples were collected using sterile syringes (Fisherbrand, Waltham, MA, USA) and then filtered through 0.45μm nylon membrane filters (Thermo Scientific Choice, Waltham, MA, USA) immediately. Based on the study of Atomssa and Gholap [42], caffeine concentrations in the solution were measured with a UV–Vis spectrophotometer (Thermo Scientific, Evolutio 60S, Waltham, MA, USA) at a wavelength of 273 nm. The caffeine removal efficiency (or rate) was determined based on Equation (1):

(1) $$\text{Removal}\;\text{Efficiency}\;(\%)=\frac{C_{0}-C_{t}}{C_{0}}\times 100$$

where $C_{0}$ is the initial concentration (mg/L) and $C_{t}$ is the final concentration after treatment (mg/L). The sample (BMCG450) with the best sorption performance was then selected as the adsorbent in the rest of the adsorption, characterization, and CMFR experiments.

Adsorption kinetics experiments were conducted by adding 50 mg of BMCG450 into 50 mL of caffeine (50 mg/L) solution in conical centrifuge tubes on the mechanical shaker. The centrifuge tubes were withdrawn from the shaker at time intervals of 0.17, 0.5, 1, 2, 4, 8, 12, 16, 24, and 36 h, respectively. Caffeine concentrations in the aqueous phase were measured as previously described. The pseudo-first-order, pseudo-second-order, Elovich, and Ritchie kinetic models, respectively, defined in Equations (2)–(5), were applied to simulate the caffeine adsorption kinetics of BMCG450.

(2) $$q_{t}=q_{e}\left(1-e^{-k_{1}t}\right)$$

(3) $$q_{t}=\frac{k_{2}{q_{e}}^{2}t}{1+k_{2}q_{e}t}$$

(4) $$q_{t}=\frac{1}{\beta }ln(\alpha \beta )+\frac{1}{\beta }lnt$$

(5) qt=qe1-e-kRt1(1-n)

where $q_{t}$ is adsorption capacity at time t(mg/g), which is calculated by the mass of caffeine sorbed in the solid phase divided by the mass of biochar; $q_{e}$ is equilibrium adsorption capacity $(\text{mg}/\text{g});k_{1}$ is the pseudo-first-order rate constant $\left({\text{h}}^{-1}\right);\;k_{2}$ is the pseudo-second-order rate constant $\left({\text{h}}^{-1}\right);\;\alpha$ is the initial adsorption rate constant (mg/g⋅h); β is the desorption constant related to surface coverage (g/mg); $k_{R}$ is the Ritchie kinetic rate constant $\left({\text{h}}^{-1}\right)$; and n is the reaction order parameter (dimensionless).

Adsorption isotherm experiments were conducted by adding 50 mg of BMCG450 into 50 mL of different dosage solutions of caffeine (5, 10, 20, 25, 50, 75, 100, 150, and 200 mg/L) in centrifuge tubes placed on the shaker. After shaking for 24 h, the samples were withdrawn to measure the aqueous caffeine concentrations. The Langmuir, Freundlich, Redlich-Peterson, and Freundlich-Langmuir isotherm models, given by Equations (6)–(9), respectively, were used to simulate the isotherm.

(6) $$q_{e}=\frac{q_{L}K_{L}C_{e}}{1+K_{L}C_{e}}$$

(7) qe=KFCe1n

(8) $$q_{e}=\frac{K_{R}C_{e}}{1+\alpha_{R}{C_{e}}^{\beta }}$$

(9) $$q_{e}=\frac{q_{FL}K_{FL}{C_{e}}^{1/n}}{1+K_{FL}{C_{e}}^{1/n}}$$

where $q_{e}$ is the adsorption capacity at equilibrium (mg/g); $q_{L}$ is the maximum monolayer adsorption capacity (mg/g); $C_{e}$ is the equilibrium concentration of the adsorbate in solution (mg/L); $K_{L}$ is the Langmuir adsorption constant (L/mg), related to the affinity of binding sites; $K_{F}$ is the Freundlich adsorption constant $(\text{mg}/\text{g})\cdot (\text{L}/\text{mg}{)}^{\text{n}}$, representing adsorption capacity; n is the adsorption intensity parameter (dimensionless), indicating the degree of favorability of adsorption; $K_{R}$ is the Redlich–Peterson isotherm constant (L/g); $\alpha_{R}$ is the Redlich–Peterson isotherm constant (L/mg); β is the exponent parameter (dimensionless), ranging between 0 and 1; $q_{FL}$ is the Freundlich–Langmuir maximum adsorption capacity (mg/g); $K_{FL}$ is the Freundlich-Langmuir adsorption constant (L/mg); and 1/n is a dimensionless exponent related to the heterogeneity of the adsorption surface.

The effect of pH on the caffeine removal efficiency of BMCG450 was determined by adding 25 mg of BMCG450 into 25 mL caffeine solutions (25 mg/L) with five different pH values (3, 5, 7, 9, and 11). The pH was adjusted using 0.1 N HCl and 0.1 N NaOH solutions. The effect of ionic strength on the caffeine removal efficiency of BMCG450 was determined by adding 25 mg of BMCG450 into 25 mL caffeine solutions (50 mg/L) with five different ionic strength values (0, 25, 50, 75, and 100 mmol/L). The ionic strength was adjusted using NaCl (Fisher Chemical, Waltham, MA, USA). All experimental conditions were the same as in the biochar screening experiment.

### 2.4. Sorbent Characterization

The Bruauer–Emmet-Teller (BET, Micromeritics ASAP2460, Norcross, GA, USA) method was used to analyze the specific surface area (SSA) of all the biochar samples. The surface morphology of the selected biochar samples (CG450 and BMCG450) was analyzed via scanning electron microscopy (SEM, Hitachi SU8020, Tokyo, Japan). The changes in the functional groups of CG450 and BMGG450 were recorded using Fourier transform infrared (FTIR, Nicolet IS 10, Waltham, MA, USA) spectroscopy and X-ray photoelectron spectroscopy (XPS, Thermo ESCALAB 250Xi, Waltham, MA, USA).

### 2.5. Caffeine Removal in CMFR

**Figure 2.**
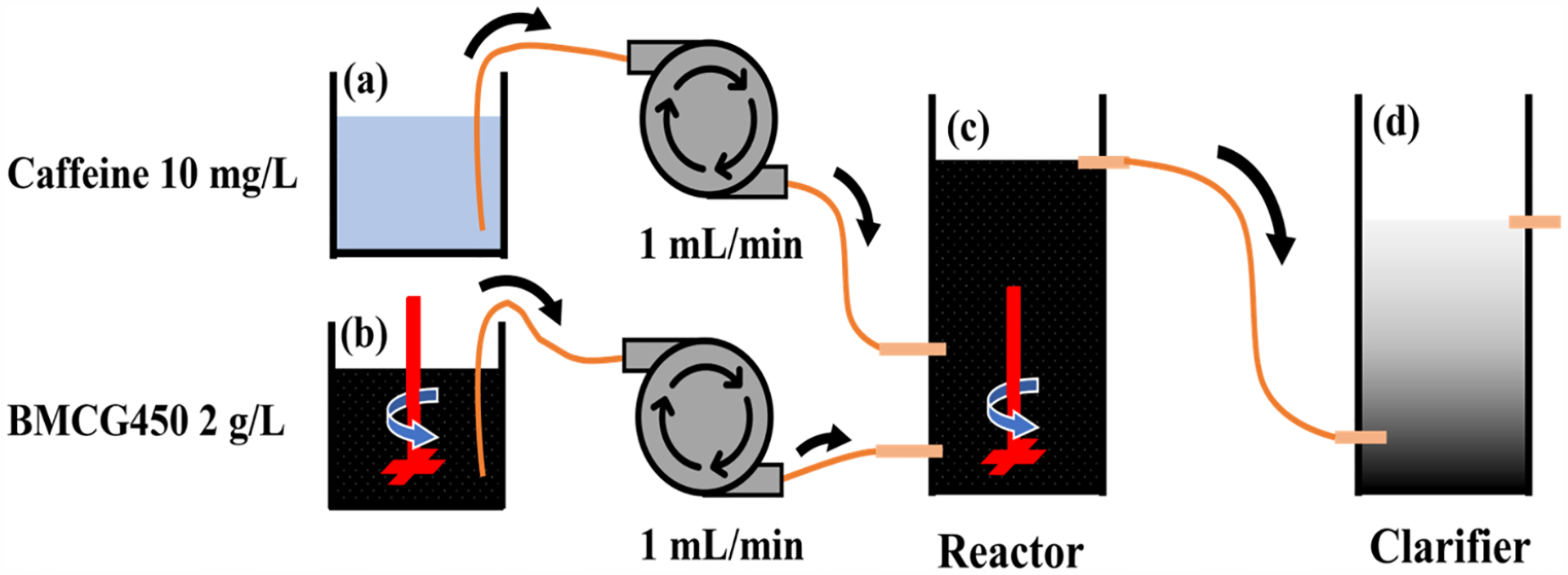
Schematic flow chart of caffeine removal in CMFR: (**a**) 10 mg/L of caffeine solution, (**b**) 2 g/L BMCG450 in DI water, (**c**) reactor where BMCG450 adsorbs caffeine, (d) clarifier where treated water is collected and spent BMCG450 settles down.

A bench-scale CMFR was constructed, as shown in Figure 2. The whole CMFR system comprised (1) a 1 L beaker (a) with 10 mg/L caffeine solution; (2) a 1 L beaker (b) where 2 g/L of BMCG450 suspension was constantly agitated with an overhead stirrer (Corning, Corning, NY, USA) to ensure homogeneous adsorbent concentrations; (3) a 750 mL reactor (c), where the adsorbent and sorbate were mixed with an overhead stirrer (Corning, USA); and (4) an 800 mL clarifier (d), where the treated solution was collected and the adsorbent settled out of the solution. Hydraulic retention time (HRT—contact time between adsorbents and sorbates in reactors), sorbate concentration, and adsorbent amount are essential factors affecting the design and performance of the reactor. To initiate the adsorption experiment in the CMFR, adsorbent and caffeine were pumped with tube pumps (Masterflex L/S, Vernon Hills, IL, USA) from reactors (a) and (b) at a flow rate of 1 mL/min. Thus, the adsorbent concentration in reactor (c) was 1 g/L, and the caffeine concentration was 5 mg/L. The mixture in reactor (c) then flowed into reactor (d). In reactor (d), the adsorbents settled down in the container, and the effluent was collected to measure the caffeine concentration.

All adsorption experiments were conducted with two replicates, with a blank as the control. Mean values are reported. The least significant difference (LSD) test was conducted to compare the difference between means at α=0.05. Experimental data were fitted to Equations (2)–(9) to reach the best model performance in terms of goodness-of-fit statistics, including the Akaike Information Criterion (AIC), sum of squared estimate of errors (SSE), and correlation between measured and modeled values $\left(R^{2}\right)$. Only R^2^ values are reported.

## 3. Results and Discussion

### 3.1. Initial Assessment

**Figure 3.**
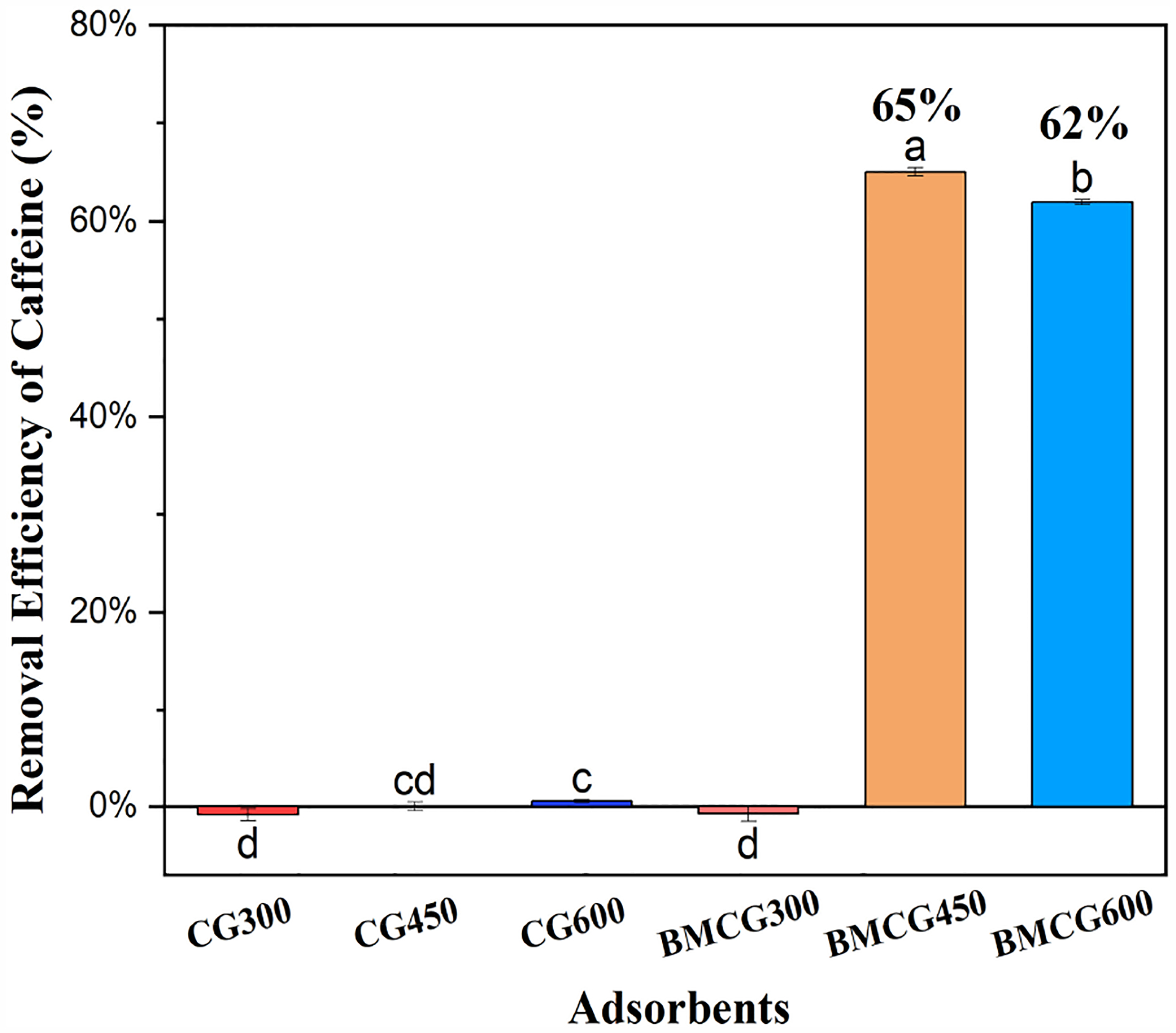
Removal efficiency of caffeine by different biochar adsorbents. Means with the same letter are not significantly different at the significance level of 0.05.

Two ball-milled biochars, BMCG450 and BMCG600, had significantly higher caffeine removal efficiencies, up to 65%, than the un-milled biochar and BMCG300, which showed almost no sorption (Figure 3). There are two significant factors that could affect the caffeine removal efficiency of the biochar: the ball-milling process and pyrolysis temperature. Ball milling can enhance biochar caffeine sorption by increasing biochar SSA and surface functional groups [43]. Moreover, the surface area of biochar generally increases with increasing pyrolysis temperature, causing the enhancement of adsorption [44]. Both CG300 and BMCG300 showed negative caffeine removal, meaning they released caffeine into the solution. This could be attributed to the incomplete carbonization of caffeine residues in SCG at 300 °C. Furthermore, after ball milling, BMCG300 looked like a sticky brown mush, confirming that it was not fully carbonized.

According to the results of this initial caffeine adsorption experiment, BMCG450 showed the most outstanding caffeine removal efficiency among the six biochar treatments. It was thus selected as the representative ball-milled SCG biochar in the follow-up experiments.

### 3.2. Properties of Adsorbents

**Figure 4.**
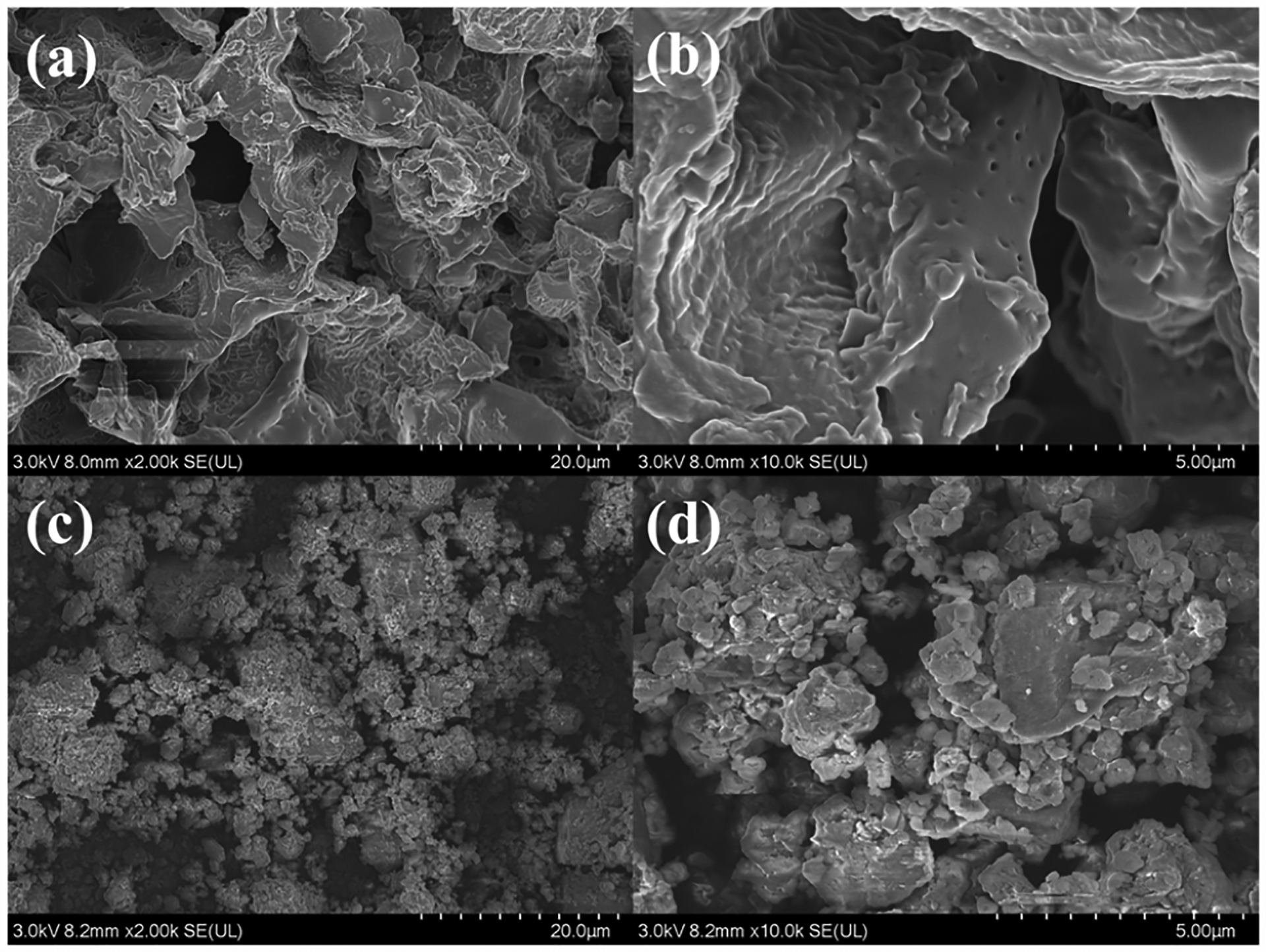
SEM images of CG450 at (**a**) 2.00k× and (**b**) 10.00k× magnifications and BMCG450 at (**c**) 2.00k× and (**d**) 10.00k× magnifications.

Ball milling, as a modification method, can crush the granular pristine biochar into ultra-fine particles with porous and rough surfaces, increasing SSA. SEM analysis confirmed the surface morphological changes on CG450 and BMCG450 (Figure 4). Moreover, the BET analysis results also confirmed that ball milling enhanced the surface areas of all biochar samples (Table 1). A roughly 167-fold increase in the surface area for CG450 and a roughly 137-fold increase for CG600 were achieved with ball milling. This BET result is, to some extent, corroborated by the initial caffeine removal assessment (Figure 3) that the two ball-milled biochars showed the highest removal efficiency of caffeine from water.

**Figure 5.**
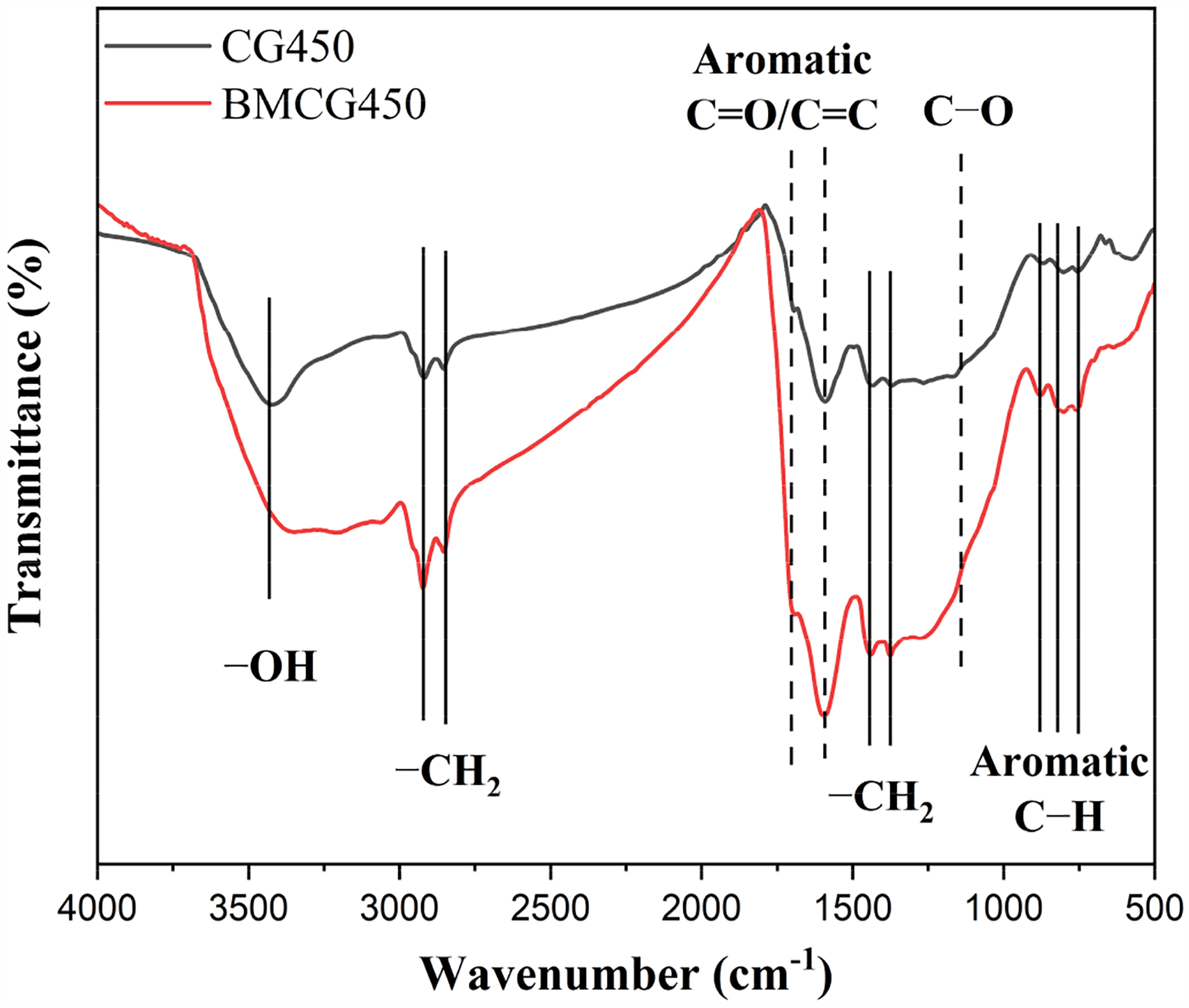
FTIR spectra of CG450 (black line) and BMCG450 (red line).

The FTIR spectroscopic analysis revealed differences in surface functional groups between CG450 and BMCG450 (Figure 5). There was no new outstanding peak showing up after the ball milling, indicating no dramatic changes in the species of the functional groups. However, there was a considerable enhancement in the amount of each functional group, including C-H (2934, 2854, 1440, and 1375 cm^−1^),C=O (1693 cm^−1^),C=C (1595 cm^−1^), C-O (1156 cm^−1^), and aromatic C-H (879, 802, and 759 cm^−1^) [45]. Therefore, compared with pristine biochar (CG450), BMCG450 had many more functional groups, especially oxygen-containing functional groups, consistent with the findings of previous studies [31,46].

**Figure 6.**
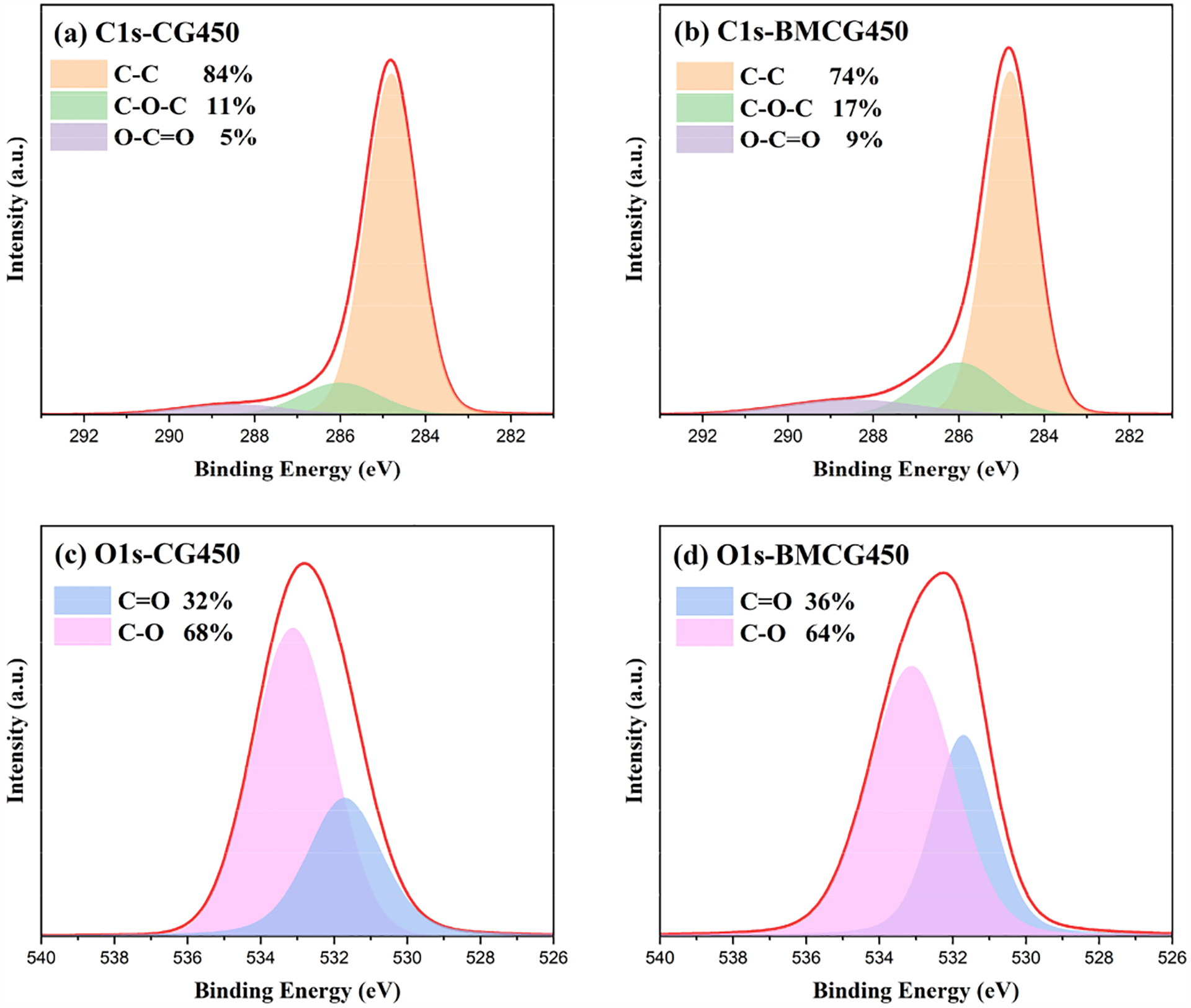
XPS spectra of (**a**) C1s-CG450, (**b**) C1s-BMCG450, (**c**) O1s-CG450, and (**d**) O1s-BMCG450.

XPS spectra analysis was used to reveal the effects of ball milling on the elemental composition and bonding state of the SCG biochars. There was a slight decrease in C1s, from 87.4% (CG450) to 84.1% (BMCG450). Meanwhile, the corresponding O1s values increased from 9.84% to 11.13% after ball milling. According to the C1s spectra in Figure 6a,b, the changes in three key spikes, C-C (284.8 eV), C-O-C (286 eV), and O-C=O (288.5 eV), in CG450 and BMCG450 indicate that ball milling enhanced the formation of oxygen-containing bonds, indicative of more oxygen-containing functional groups on the ball-milled biochar surface. Moreover, the O1s spectra in Figure 6c,d of the two samples suggest that ball milling might increase the proportion of carboxyl functional groups (C=O, 531.71 eV), but reduce the proportion of hydroxyl groups (C-O, 533.12 eV). In comparison to hydroxyl groups, carboxyl groups are more easily dissociated and more prone to chemical reactions with caffeine in water.

### 3.3. Caffeine Adsorption Kinetics and Isotherms

**Figure 7.**
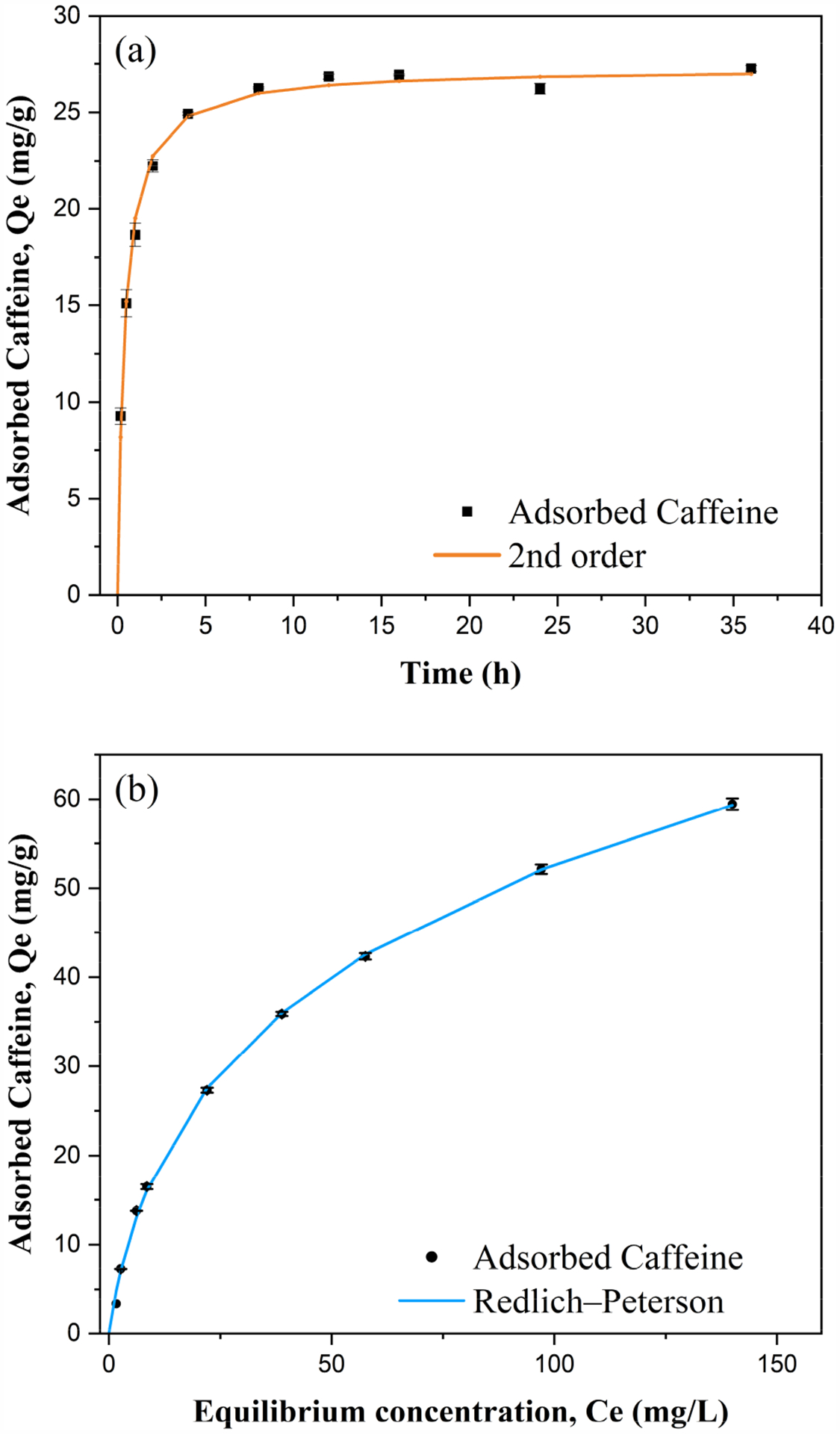
Kinetics (**a**) and isotherms (**b**) of caffeine adsorption onto BMCG450. Symbols represent experimental data and lines represent model results.

Establishing the adsorption kinetics is necessary for us to understand how quickly BMCG450 reaches equilibrium (equilibration time), providing insights into the mechanisms governing the adsorption process. Figure 7a shows that caffeine adsorption on BMCG450 increased dramatically in the first two hours, then attained equilibrium within eight hours. At the equilibrium, caffeine adsorption reached about 27.26 mg/g. The pseudo-first-order, pseudo-second-order, Elovich, and Ritchie kinetic models were applied to simulate the caffeine adsorption kinetics of BMCG450 (Table 2). Among them, the pseudo-second-order kinetic model aligned the best with the experimental data $\left({\text{R}}^{2}=0.996\right)$, indicating that the caffeine adsorption onto BMCG450 could be a heterogenous chemisorption process affected by multiple factors, such as surface area and functional groups [47]. Considering that the Ritchie kinetic model $\left({\text{R}}^{2}=0.995\right)$ also provided a strong fit, this suggests that while chemisorption is the dominant mechanism (as indicated by the pseudo-second-order model), additional physical interactions such as surface diffusion or van der Waals forces may also contribute to the overall adsorption process. This implies that the adsorption of caffeine onto BMCG450 is governed by a combination of mechanisms rather than a single dominant process.

Adsorption isotherms show the relationship between initial sorbate concentration and the adsorbent capacity of the sorbent. The adsorption capacity of BMCG450 for caffeine increased as the caffeine initial concentration increased (Figure 7b). The Langmuir, Freundlich, Redlich–Peterson, and Freundlich–Langmuir isotherm models were used to simulate the isotherm (Table 2). According to the Langmuir model, the maximum caffeine adsorption of BMCG450 was about 82.65 mg/g, higher than that of many other carbonaceous adsorbents in the literature [29,48,49]. The Freundlich–Langmuir isotherm model gives an even higher maximum sorption capacity of 100.44 (mg/g). Among all the models, Redlich–Peterson and Freundlich–Langmuir provided the best fit (${\text{R}}^{2}=0.999$), indicating that caffeine adsorption onto BMCG450 is governed by a combination of physical and chemical adsorption mechanisms. The high specific surface area and surface functional groups of BMCG450 facilitate multiple interactions, including Van der Waals forces, π-π stacking, hydrogen bonding, electrostatic attraction, and surface complexation. The strong fit of these hybrid models further supports the presence of both monolayer and multilayer adsorption processes, making BMCG450 an effective adsorbent for caffeine removal from water.

### 3.4. Effect of pH on Caffeine Adsorption

**Figure 8.**
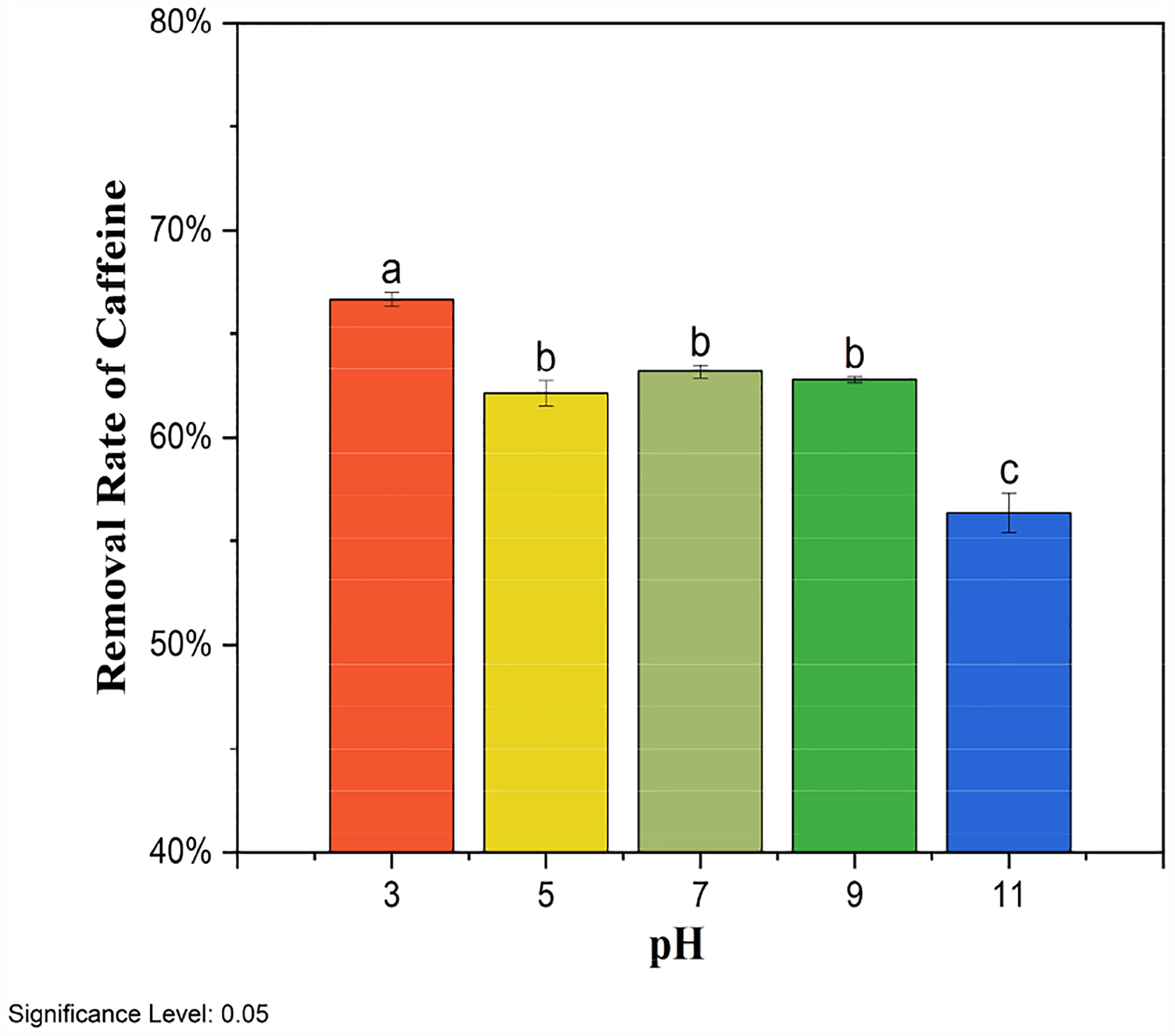
Effect of pH on the removal of caffeine by BMCG450. Means with the same letter are not significantly different at the significance level of 0.05.

Solution pH plays an essential role in the adsorption of organic pollutants onto biochar because pH can influence the charge of the biochar surface, as well as the degree of ionization of organics in the solution. Here, the pH effect was investigated by changing the initial solution pH from 3 and 11, and the corresponding caffeine removal rates by BMCG450 are shown in Figure 8. Generally, as pH increased, caffeine removal by BMCG450 decreased. The caffeine removal rate by BMCG450 slightly decreased when the pH increased from 3 to 5, followed by a relatively stable phase in the pH range of 5 to 9. However, as pH rose to 11, the caffeine removal rate showed significant decrease.

Caffeine has a pK_a_ value of 8.3 [50]. Beltrame, Cazetta, de Souza, Spessato, Silva, and Almeida [30] pointed out that neutral-form caffeine dominates when solution pH is below 5.5. The anionic form of caffeine exists in solutions with a pH above 5.5. After pH exceeds 8.3, the anionic form of caffeine with a negative charge becomes the dominant species. Meanwhile, with the pH change, BMCG450’s surface charge was also affected. The pH_pzc_, the pH at which the net charge of the adsorbent surface is zero [51], may also provide some insight. According to the study of Nguyen et al. [52], the pH_pzc_ of SCG biochar pyrolyzed at 500 °C is about 7.25.

At low pH < 5 in this study, the BMCG450 surface was protonated, and caffeine existed in the neutral form. They may interact with each other through mechanisms such as hydrogen bonding and π-π stacking [53]. This leads to effective adsorption, though a slight decline is observed as pH increases from 3 to 5. In the pH range of 5 to 9, some caffeine molecules become anionic, but the BMCG450 surface remains partially protonated, maintaining the electrostatic attraction between negatively charged caffeine species and positively charged biochar sites. However, some hydrogen bonding interactions may weaken. At a pH of 11, caffeine existed predominantly in the anionic form, and the surface of BMCG450 mainly carried negative charges due to deprotonation. Electrostatic repulsion between the negatively charged caffeine and biochar surface reduces adsorption efficiency.

### 3.5. Effect of Ionic Strength on Caffeine Adsorption

**Figure 9.**
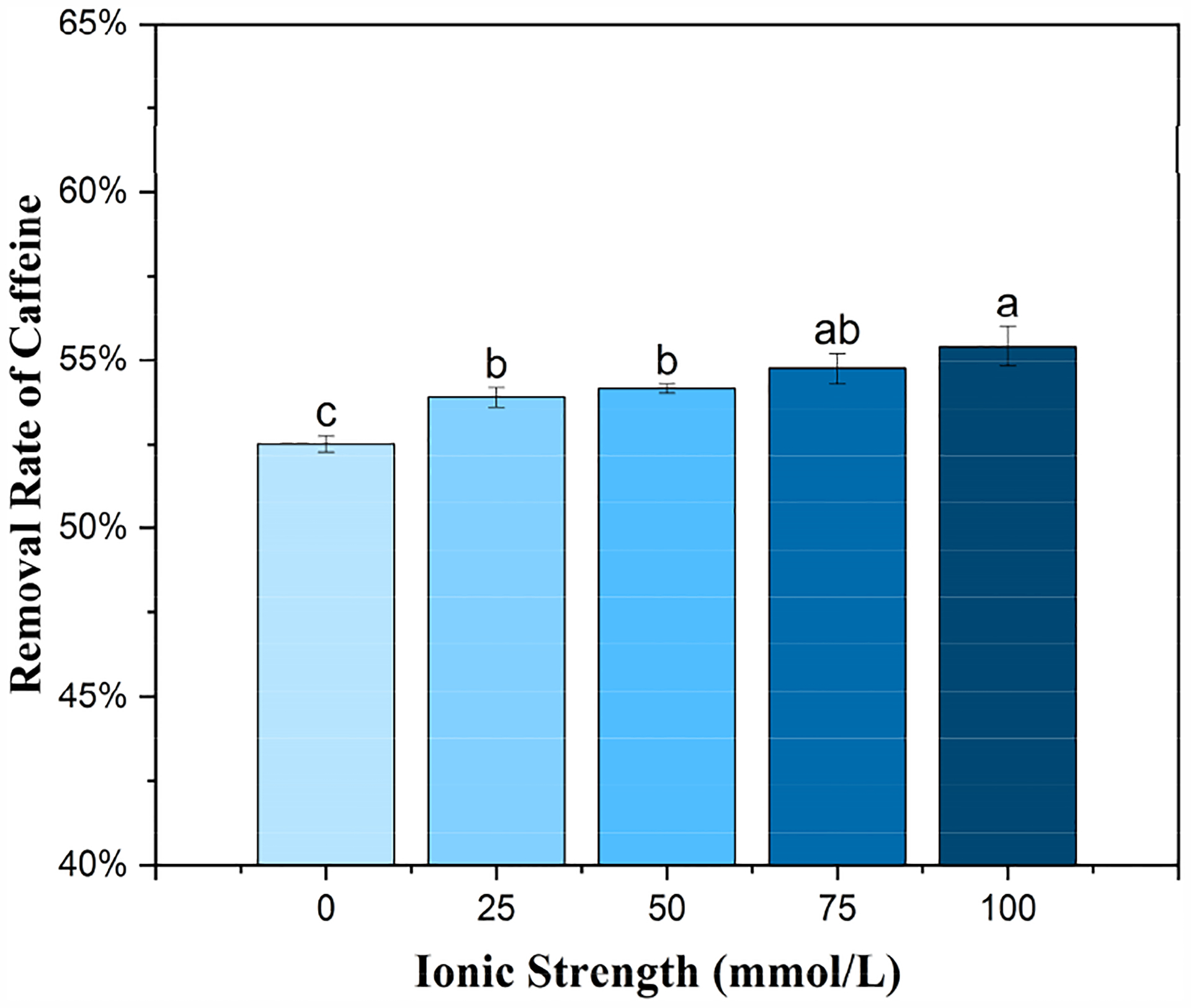
Effect of ionic strength on the removal of caffeine by BMCG450. Means with the same letter are not significantly different at the significance level of 0.05.

The adsorption of caffeine by BMCG450 slightly increased as NaCl concentrations increased from 0 to 100 mmol/L (Figure 9). There are conflicting reports on the impact of solution ionic strength on adsorption, depending on specific circumstances. Wang et al. [54] found that ionic strength had no significant impact on ciprofloxacin adsorption onto activated carbon. However, Couto Jr, Matos, da Fonseca, Arroyo, da Silva, and de Barros [53] found that ${\text{Ca}}^{2+}$ and ${\text{mg}}^{2+}$ in solution decreased the caffeine adsorption of activated carbon due to a competition effect. Some reports have suggested that increasing ionic strength could suppress the adsorption of organic chemicals onto carbon nanotubes due to the formation of a highly compacted structure, the so-called squeezing-out effect [55–57]. Liu et al. [58] found that increasing NaCl and CaCl_2_ concentrations enhanced the sorption of ketoprofen and considered that this resulted from the electrostatic screening effect by the cations. Similarly, the increased adsorption of caffeine on BMCG450 with increasing ionic strength could result from the electrostatic screening effect.

### 3.6. Caffeine Adsorption in CMFR

**Figure 10.**
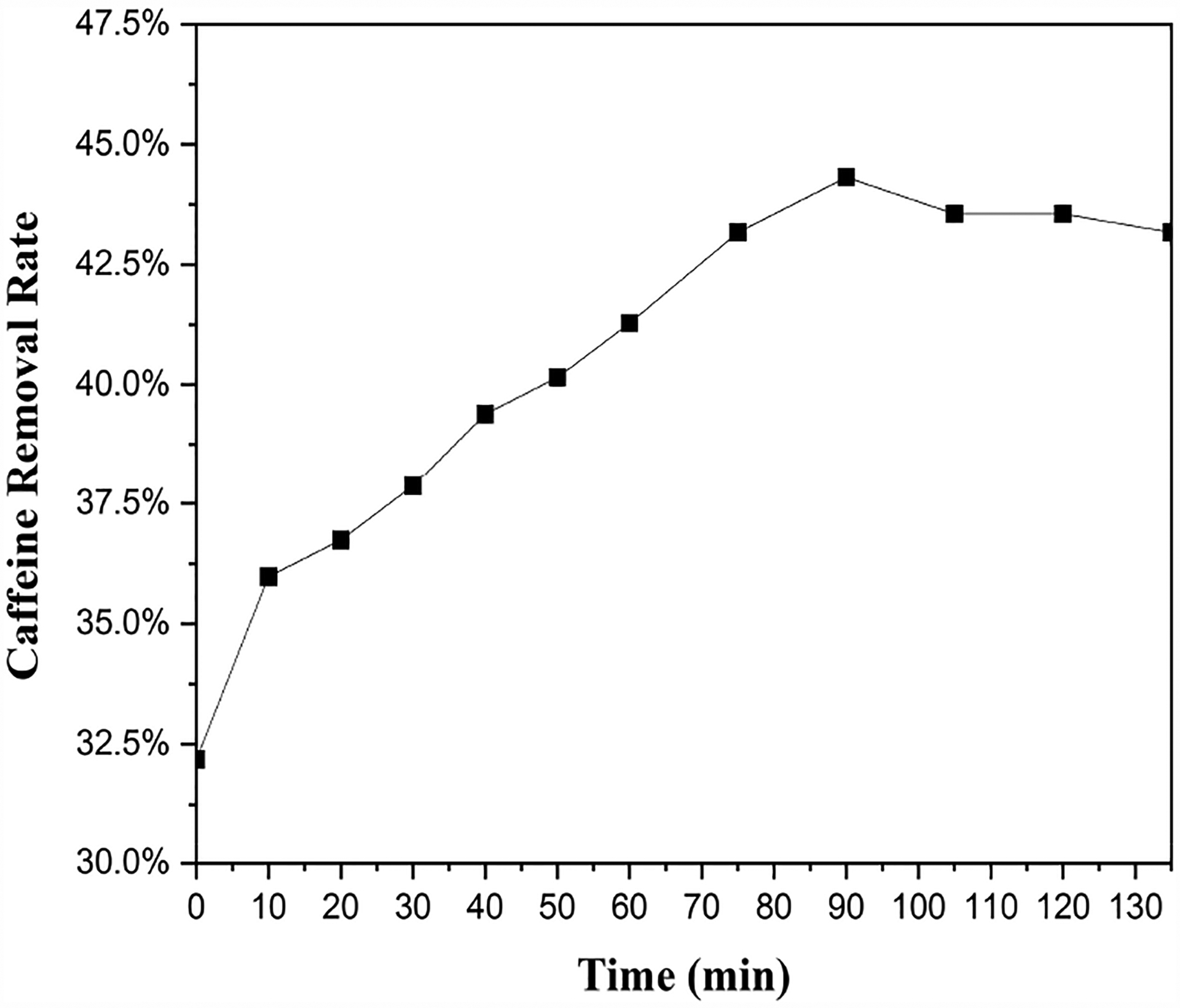
Caffeine removal rate of BMCG450 in a completely mixed flow reactor (CMFR) under continuous flow conditions.

Caffeine removal by BMCG450 under continuously flowing conditions was determined in the CMFR. During the operation, the removal rate of caffeine by BMCG450 increased with time until the reaction system reached equilibrium and stability after about 90 min, and then the removal rate remained at about 43% (Figure 10). There was no concentration breakthrough during the experiment, indicating that BMCG450 effectively removed caffeine from the water. This removal efficiency can be further enhanced by adjusting the caffeine concentration, BMCG450 dosage, or flow rate. The results from the CMFR indicated that BMCG450 can serve as an effective adsorbent under continuous flow conditions for the fast and convenient removal of caffeine from water.

### 3.7. Practical Considerations

This study shows that ball-milled SCG biochar is a promising adsorbent for environmental applications. Pyrolysis temperature and engineering modifications are key practical considerations that need to be factored in the production of biochar derived from spent coffee ground. In the literature, a magnetic activated SCG biochar produced at a pyrolysis temperature of 700 °C was reported for the removal of ibuprofen from ground water and lakes [59]. These authors indicated that the simultaneous magnetization and activation of the biochar was an effective approach to improving the adsorption capacity of ibuprofen. In another study, SCG biochar produced at a pyrolysis temperature of 450 °C was modified by phosphoric acid and mercaptoacetic acid [60]. This biochar exhibited an impressive ${\text{Cd}}^{2+}$ adsorption capacity of 205 mg/g. An excellent sorption of norfloxacin at the maximum absorption capacity of 69.8 mg/g was also noted with SCG biochar produced at a pyrolysis temperature of 500 °C [52]. This biochar was pristine without modification and the optimal pyrolysis temperature was found through testing pyrolysis temperatures of 300 °C, 500 °C, 700 °C, and 900 °C. In addition to its application in water treatment, biochar derived from spent coffee grounds produced at pyrolysis temperatures of 400–600 °C was tested for the capture of carbon dioxide from flue gas [61]. Biochar produced at 600 °C performed the best. Clearly, the selection of modification methods, if needed, for biochar derived from spent coffee grounds is dependent on the specific contaminant of concern. Ball milling, as shown in our study, is an effective physical modification method that significantly increases the SSA and functional groups of biochar. Our study and the existing literature generally support an optimal pyrolysis temperature range of 450–600 °C.

Another consideration for the practical application of caffeine removal is the maximum caffeine adsorption capacity of the biochar. For BMCG450 in this study, the maximum caffeine adsorption capacity reached 82.65 mg/g, as obtained by the Langmuir model (Table 2). This maximum adsorption capacity can be compared with several adsorbents that were tested for caffeine removal. A MgAl-LDH/biochar composite using bovine bone biochar as a support for Layered Double Hydroxide (1:2) nanoparticles exhibited a maximum adsorption capacity of 26.22 mg/g at 40 °C [62]. These authors noted that physical adsorption was the dominant mechanism, possibly relating to the layered nature of LDH and the decreased sorption as temperature increased. A palm-activated carbon had a Langmuir maximum adsorption capacity of only 8.50 mg/g at 30 °C [63]. These authors did not report the specific surface area of the sorbent, yet they stated that chemisorption was the dominant adsorption mechanism, and the Langmuir maximum adsorption capacity did not change much with temperature. A thermally modified bentonite (at 400 °C) had a Langmuir maximum adsorption capacity of 80.3 mg/g [64]. Bentonite is a 2:1-type clay, typically featuring a temperature-dependent specific surface area (around 100–200 m^2^/g). The thermal modification contributed to the large adsorption capacity, likely in association with the increased SSA. The highest value reported in the literature is 367.2 mg/g from a grape stalk biochar, which exhibited an exceptionally high specific surface area of 1100 m^2^/g [65]. The literature generally supports that the maximum caffeine adsorption capacity is a valid engineering parameter for the design of adsorption systems. Sorbents offering both physical and chemical sorption are preferred, and BMCG450—examined in this study—possesses such properties with reasonably high adsorption capacity for practical applications.

The final practical consideration is the recovery and reusability of the ball-milled SCG biochar. Through not experimentally examined in this study, scaling up the system shown in the CMFR is critically important to assess its cost-effectiveness. Note that ball-milled SCG biochar has an extremely fine particle size and high dispersibility, meaning that it easily flows with water. This can potentially lead to significant loss of the adsorbent if not controlled well. The recovery and reuse of ball-milled biochar in flowing water systems remain critical challenges that need to be addressed, currently limiting its large-scale practical application. Thermal, solvent, and magnetic methods are common regeneration methods [66] and deserve our future examination. Nevertheless, given SCG is a byproduct of the coffee production process and the growing concerns about caffeine in aquatic environments, this study offers a technical basis for developing a sustainable and circular solution by integrating waste reuse with pollutant removal.

## 4. Conclusions

This study demonstrates that ball-milled SCG biochar is an excellent adsorbent for the removal of caffeine from water. Ball milling dramatically increased the SSA of biochar by up to 168 times. Meanwhile, FTIR and XPS analyses revealed more oxygen-containing functional groups on the surface of the biochar after the ball milling. All these could be factors contributing to the increased adsorption of caffeine onto ball-milled SCG biochar. The Langmuir maximum caffeine adsorption capacity of BMCG 450 reached 82.65 mg/g. Both solution pH and ionic strength affected caffeine removal by BMCG450. As pH increased, caffeine adsorption onto the biochar decreased due to the increase in electrostatic repulsion. However, caffeine adsorption was stable in the pH range of 5 to 9. Increases in ionic strength slightly enhanced caffeine adsorption, possibly due to the electrostatic screening effect of cations. BMCG450 also showed good performance in a CMFR under continuous flow conditions. All of these findings indicate that BMCG450 biochar can be used as a low-cost and efficient adsorbent for the removal of caffeine from water under practical conditions. The regeneration and reusability of biochar will be a key focus for further exploration.

## Figures and Tables

**Table 1.** BET specific surface area of biochar produced at three pyrolysis temperatures before and after ball milling.

Biochar	SSA $\left(m^{2}/g\right)$ for CG	SSA $\left(m^{2}/g\right)$ for BMCG	Magnification
${300\;}^{\circ }\text{C}$	0.190	1.042	5.48×
${450\;}^{\circ }\text{C}$	0.060	10.114	168.67×
${600\;}^{\circ }\text{C}$	0.147	20.138	136.99×

**Table 2.** Best-fit adsorption models and their parameters, as defined in Equations (2)–(9).

Kinetics Model	Parameter 1	Parameter 2	Parameter 3	$R^{2}$
Pseudo-first-order	$k_{1}=1.574\;(1/\text{h})$	$q_{e}=25.953\;(\text{mg}/\text{g})$		0.967
Pseudo-second-order	$k_{2}=0.09204\;(1/\text{h})$	$q_{e}=27.279\;(\text{mg}/\text{g})$		0.996
Elovich	β=0.000000304(g/mg)	α=751.413(mg/g/h)		0.959
Ritchie	$k_{R}=0.000220\;(1/\text{h})$	$q_{e}=27.103\;(\text{mg}/\text{g})$	1/n=1.912	0.995
Isotherm Model	Parameter 1	Parameter 2	Parameter 3	$R^{2}$
Langmuir	$K_{L}=0.020\;(\text{L}/\text{mg})$	$q_{L}=82.651\;(\text{mg}/\text{g})$		0.983
Freundlich	$K_{F}=5.964\;(\text{mg}/\text{g})\cdot (\text{L}/\text{mg}{)}^{\text{n}}$	n=0.473		0.990
Redlich-Peterson	$K_{R}=3.849\;(\text{L}/\text{g})$	$\alpha_{R}=0.2159\;(\text{L}/\text{mg})$	β=0.733	0.999
Freundlich-Langmuir	$K_{FL}=0.0389\;(\text{L}/\text{mg})$	$q_{FL}=100.442\;(\text{mg}/\text{g})$	1/n=0.728	0.998

## Data Availability

Data available on request due to restrictions, e.g., privacy or ethical. The data presented in this study are available on request from the corresponding author.
